# Spatial risks of *Orthoebolavirus* spillover vary based on outbreak type

**DOI:** 10.1016/j.ijid.2025.108180

**Published:** 2025-11-03

**Authors:** Mekala Sundaram, Patrick R. Stephens

**Affiliations:** 1 Savannah River Ecology Laboratory, University of Georgia, Athens, USA; 2 Department of Infectious Diseases and Center for Precision One Health, University of Georgia, Athens, USA; 3 Department of Integrative Biology, Oklahoma State University, Stillwater, USA

**Keywords:** Spillover risk, Emerging infectious diseases, Ebolavirus disease, Zaire ebolavirus, Sudan ebolavirus, Machine learning

## Abstract

**Objectives:**

We develop and test a risk map for Orthoebolaviruses which are emerging infectious pathogens primarily concentrated in sub-Saharan Africa. The accuracy of predictive models and risk maps has been limited thus far by uncertainty in mechanisms underlying spread, low number of known outbreaks, and in how well various drivers predict different types of outbreaks (e.g. human vs epizootic outbreaks, and outbreaks of different viral species).

**Methods:**

Here, we explore frugivory and other factors as mechanisms of *Orthoebolavirus* spread and demonstrate statistical methods with repeated cross-validation that can be used even with very small data sets to explore how different factors influence different classes of events using ensemble machine learning logistic regression.

**Results:**

We show that covariates predicting outbreaks with the highest discrimination power are frugivore richness (area under curve [AUC] = 0.95) and fruit tree (*Ficus)* habitat suitability (AUC = 0.94). We found that *Ficus* distributions contributed to predictions of past *Orthoebolavirus* outbreaks relatively equally, regardless of type, based on feature contributions estimated using Shapley value calculations. In contrast, frugivore richness was a better contributor of predictions of epizootic than human outbreaks. Hunting activity was a poor predictor overall (AUC = 0.85) but contributed to some Sudan outbreaks predictions.

**Conclusions:**

Our results suggest that different drivers best influence different classes of *Orthoebolavirus* outbreaks and models taking into account a variety of factors are needed to predict future spillover events.

## Introduction

Orthoebolaviruses are infectious agents from the family Filoviridae causing outbreaks primarily in sub-Saharan Africa. In total, four species of human pathogenic viruses are known, including Zaire ( *Orthoebolavirus zairense*), Sudan ( *O. sudanese*), Bundibugyo ( *O. bundibugyoense*), and Taï Forest ( *O. taiense*, formerly, Ivory Coast ebolavirus) orthoebolaviruses, all of which have infected human populations and most of which have caused hemorrhagic fevers that are fatal [1,2]. The case fatality rates of known outbreaks are relatively high ( *>*30% [3]), and attempts to map spillover risk to date [4,5] have been hampered by a lack of clarity about mechanisms underlying spillover. One particular challenge is that the primary biological hosts have not been definitively identified [6–8], although recent work links specific groups of species to spillover events [9–11]. Risk maps have also generally treated *Orthoebolavirus* outbreaks monolithically and have not directly investigated whether different factors might better predict different classes of outbreaks. For example, the most influential drivers might differ slightly between human spillover events and epizootic outbreaks, among different species of virus, or in different regions. In addition, key variables such as the presence of primary vs secondary amplifying wild hosts and habitat conditions that facilitate transmission among frugivorous mammals (examined in later sections) have not been incorporated into *Orthoebolavirus* spillover risk models and maps.

A number of studies have used machine learning and other statistical approaches to map spillover and outbreak risk of orthoebolaviruses (by which we refer to the four human pathogenic species here and exclude *O. restonense* and *O. bombaliense*, which are not known to cause disease in humans) based on environmental and ecological factors [5,12]. However, recent studies [13–17] have yielded important ecological insights that have not yet been reflected in risk maps and have also raised a number of important questions about what factors will most accurately predict spillover risk. For example, in the first comprehensive study of the reservoir potential of all African mammals, Sundaram et al. [15] used ensemble machine learning methods to show which species of mammals were most likely to be exposed to orthoebolaviruses in the wild and which species would be most likely to survive infection if exposed. They defined species that are likely to be infected in the wild and tolerate infections well (i.e. show little or no mortality when infected) as potential primary hosts. They contrasted these species with others that are also likely to be infected by these viruses in the wild but that tend to experience high mortality when infected, which they defined as likely secondary amplifying hosts. Sundaram et al. [15] showed that all African species in several bat clades (particularly, frugivorous pteropodid bats but also some insectivore clades) fit the profile of a primary host, whereas all African representatives (including humans) of several primate clades better fit the profile of a secondary amplifying hosts. Intuitively, it would be expected that the diversity of primary hosts that tolerate infections well enough to spread them widely would be the most important factor affecting spillover risk. However, studies that have investigated the influence of mammalian host biodiversity on *Orthoebolavirus* spillover risk (e.g. [4,5,9]) have not generally distinguished between likely primary and secondary amplifying hosts; therefore, which of these groups of species best predict spillover risk is unclear.

A follow-up study, Sundaram et al. [16], compared more than a dozen path diagrams reflecting different hypotheses about the factors that proximately vs more indirectly affect spillover risk. Surprisingly, this study showed that the best supported path diagrams were ones in which the diversity of fruit-eating species in general, including fruit bats (primary hosts) and cercopithecid primates (secondary hosts), directly influenced spillover risk. Counterintuitively, pteropodid bat diversity affected risk only indirectly through influencing the overall diversity of frugivorous mammals. Sundaram et al. [16] also showed strong evidence that *Ficus* habitat suitability directly influences spillover risk and that environmental factors, such as temperature and rainfall [5], affect spillover risk only indirectly through their influence on *Ficus*. These results are also consistent with several previous studies (e.g. [9,18,19]). However, so far, neither frugivorous mammal diversity (sensu lato, including all endemic to Africa) nor *Ficus* habitat suitability have been directly incorporated into *Orthoebolavirus* spillover risk maps.

Another limitation of previous studies is that few have attempted to compare the fit of risk maps based on different ecological factors. For example, previous studies that have mapped risk based on host biodiversity have tended to make a case for some set of hosts being important and simply built maps using those sets of species (e.g. [4,5], but see [9]) rather than comparing the fits of maps based on a variety of candidate species. Studies have also not generally considered whether different factors might better explain outbreaks caused by different species of orthoebolaviruses (e.g. *Zaire* vs *Sudan orthoebolavirus*), outbreaks that occur in different regions (e.g. wet tropical regions of central Africa vs drier regions to the east where outbreaks have also been documented), or epizootic events (e.g. [20]) vs spillover into human populations (reviewed in [21]). For example, Schmidt et al. [5] included many epizootic events in their analyses but did not test whether different factors better explained human vs epizootic spillover. The only previous study that we are aware of to evaluate the fit of a spatial model of spillover risk to different classes of Orthoebolavirus outbreaks, the study by Olivero et al. [9], showed that their final model predicted *Zaire orthoebolavirus* spillover better than it predicted spillover of *Sudan Orthoebolavirus* . However, even this study did not directly test whether models based on other factors better predicted spillover of the latter species. To summarize, *Orthoebolavirus* spillover risk models, thus far, have not included primary vs secondary hosts, accounted for outbreak type (e.g. human vs epizootic, or by virus species), or compared the predictive power of different ecological drivers. Key factors such as *Ficus* habitat suitability remain untested, limiting understanding of what drives distinct outbreak types. Our study seeks to fill in these key gaps.

Here, we generate risk maps using many different sets of hosts and other eco-environmental factors to see which most accurately predict *Orthoebolavirus* spillover risk in aggregate. For the first time, we also examine which factors best predict different classes of outbreaks: spillover of different viral species and epizootic vs human spillover events. Our results suggest that different factors best predict patterns of spillover risk depending on the viral species, region, and type of spillover event that is considered. The methods we use to explore how well various factors predict individual disease events have also infrequently been applied in previous studies of spillover risk or disease dynamics in general and could prove useful in other emerging and relatively rare disease systems.

## Methods

### Outbreak data

We used outbreak data from the study by Sundaram et al. [16], which georeferenced all epizootic and zoonotic human outbreaks of Orthoebolavirus documented up until 2021 (see Sundaram et al. [16] for additional details on sources from which data were compiled and how spillover events were georeferenced). We joined spillover events with a 50- × 50-km grid cell layer spread across Africa, matching the resolution to aggregated continuous predictor variables. This is similar to the resolution used in previous studies mapping spillover and outbreak risk (e.g. [5,9,22,23]).

### Predictive covariates

We gathered predictive layers from multiple sources and aggregated all variables to 50- × 50-km grid cell resolution. We downloaded mammal ranges from the International Union for Conservation of Nature and Natural Resources [24]. Using “spatial join” in ArcGIS Pro 3.2.2 [25], we overlaid a 50 × 50 km grid over range shapefiles and calculated species richness of all mammals, as well as the diversity of all African primates, bats, and artiodactyl (even-toed hoofed mammal) families: Cercopithecidae, Pteropodidae, Vespertilionidae, Rhinolophidae, Hipposideridae, Molossidae, Hominidae, Nycteridae, Cervidae, and Bovidae. The richness of fruit-eating mammals as a group can also be important in driving outbreaks [15,16]; therefore, we gathered diet information for all African mammals from EltonTraits [26]. We used these data to calculate the richness of all mammals with 60% or more of their diet composed of fruit. We also computed the richness of mammals with 60% or more of their diet composed of plants, seeds, and scavenging. Sundaram et al. [15] also estimated infection statuses and host statuses of all African mammals to orthoebolaviruses. We merged these predictions with geographic ranges of mammals in 50- × 50-km grid cells to calculate the richness of species that are expected to be exposed in the wild and susceptible to infection but experience high mortality when infected, hereafter referred to as secondary hosts. We also computed richness of species that are likely to be infected in the wild and to experience little if any mortality when infected, hereafter referred to as reservoirs. We also computed average *Ficus* habitat suitability and species richness of *Ficus* species georeferenced and published in the study by Sundaram et al. [16]. We merged *Ficus* species ranges with species fruit volumes using data and data sources outlined in the study by Sundaram et al. [16] and computed the average fruit volumes for each grid cell. Bioclimatic variables might also play a role in emergence of *Orthoebolavirus* [5], so we gathered average mean annual temperature, annual precipitation, temperature seasonality, and precipitation seasonality for each grid cell using WorldClim [27]. Finally, we downloaded a recently published GIS layer of wild animal hunting activity for Africa [28]. We computed average hunting activity in each grid cell across Africa.

#### Statistical models.

Using R package “caret” [29], we created risk maps by fitting ensemble machine learning logistic regressions using a presence pseudo-absence approach where previous sites of *Orthoebolavirus* spillover (1) were compared with randomly chosen sites with no documented spillover (0). Because the primary purpose of the analysis was to assess whether different covariates more effectively predicted specific outbreak types, each model was first constructed using a single predictive covariate. Next, we ranked the importance of each predictive variable based on their individual area under the curve (AUC) values in discriminating areas of spillover vs no spillover. To assess accuracy and avoid model overfit, we used downsampling of pseudo-absence localities to balance outcomes and performed 1000 repeats of 10-fold cross-validation. We used the “resamples” function from the R package caret to aggregate, analyze, and summarize model performances across repeated cross-validation model runs, yielding ranges for AUC values, specificity, and sensitivity metrics. We chose the co-variates with highest AUC values in discriminating spillover vs non-spillover areas and created a final model to evaluate importance in predicting each past *Orthoebolavirus* outbreak and for developing a predictive risk map. We also included the hunting activity covariate to explore in which outbreaks human exposure via hunting may have played an important role and developed a predictive risk map for this layer because epidemiologists and health care workers have frequently described hunting as an important trigger of past outbreaks [21].

We estimated the importance of the top two biological co-variates and hunting activity variable in predicting each *Orthoebolavirus* outbreak using Shapley values and leave-one-out cross-validation (LOOCV) methods. Shapley values are a metric borrowed from game theory, used to quantify “payout” of each predictor variable in predicting individual observations [30]. For our global model of *Orthoebolavirus* predicted by frugivore richness, *Ficus* suitability, and hunting activity, Shapley values were computed to reflect contributions of predictors toward each outbreak prediction. We used R package “iml” to calculate Shapley values [30]. This allowed us to compare the importance of predictors in different classes of outbreaks. Another comparable approach is the LOOCV method, with models fit to individual covariates. For each covariate, we dropped a single row from our list of outbreaks, fit an ensemble logistic regression model with R package caret, and repeated cross-validation (10 folds, 1000 repetitions) and predicted probability of spillover for the dropped row. Using this approach, we gathered the predicted probability of risk and, thus, importance of each individual covariate in predicting each known outbreak. Because these results were qualitatively similar to Shapley values contributions, we present LOOCV results in the Supplementary Materials.

Using these models, we created risk maps of *Orthoebolavirus* spillover. We predicted the risk of *Orthoebolavirus* from models for the top two covariates and hunting of wild animals for each 50- × 50-km grid cell in Africa because analyses showed that each type of outbreak and outbreaks in all regions we considered were predicted with high accuracy by at least one of these variables. We also visualized the differences and concurrences in predictions of relative risk from the top two covariates using the bivariate function of ArcGIS Pro. Finally, we created risk maps based on each individual covariate to allow their predictions to be more easily compared, which we include in the online supplementary materials. We compared our predictive risk maps with most recent *Orthoebolavirus* outbreaks from 2022 to present (see the table in Supplementary Materials for details of three additional outbreaks recorded since 2021). Georeferencing of outbreak sources was performed based on case reports from published literature sources, public health organizations such as the World Health Organization, and Africa Centers for Disease Control and Prevention.

## Results

Multiple variables had a very high accuracy in machine learning models predicting spatial occurrence of *Orthoebolavirus* outbreaks (n = 44 human outbreaks with 31 unique geographic co-ordinates and n = 17 animal outbreaks with 12 unique geographic coordinates), with mean AUC >0.85 and even the lower limit AUC from the interval of AUC values estimated through repeated cross-validation remaining above 0.70 (Table 1). This suggests consistent good model performance across model runs trained on different subsets of data. Among all tested variables, richness of frugivores ( >60% of diet including fruit) was the best overall predictor of outbreaks, with highest mean AUC value of 0.95 (range of AUCs is 0.79-0.998, Table 1). Other dietary groups, such as plant consumption and scavenging, performed poorly compared with frugivore, with mean AUC < 0.8 and range of AUCs overlapping 0.5, suggesting no discriminative ability between areas of spillover and no spillover (Table 1). Frugivore richness was followed by richness of species from the family Cercopithecidae and *Ficus* habitat suitability, both with mean AUCs of 0.948 (range 0.84-0.99) and 0.940 (range 0.87-0.99, Table 1). Wild animal hunting activity did not predict *Orthoebolavirus* outbreaks as well as these covariates but still had an AUC score of 0.870 (range 0.6-0.99, Table 1). Environmental variables, diversity of most mammalian families, reservoir status, and secondary host status did not perform as well as other covariates in predicting outbreaks, with comparatively low mean AUC values (Table 1).

**Figure 1.**
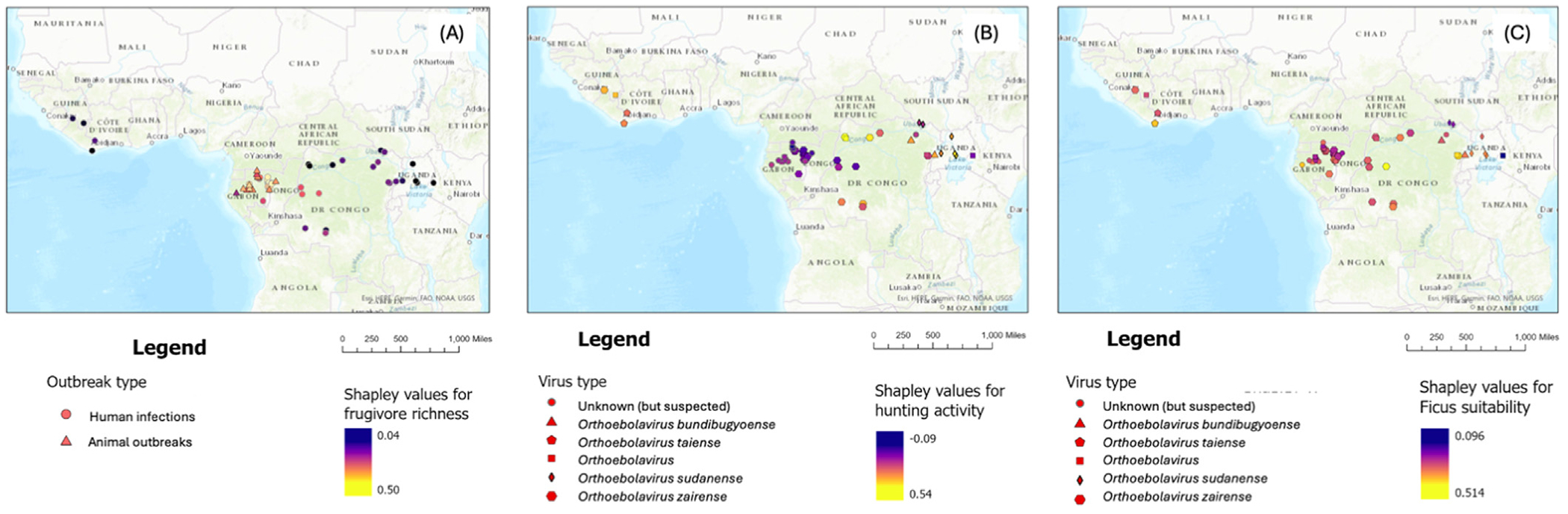
Contributions of frugivore richness (a), wild animal hunting activity (b) and *Ficus* habitat suitability (c) in explaining past *Orthoebolavirus* outbreaks. Predictor contributions to each individual outbreak were computed using Shapley values, with lighter colors (yellow) indicating that a given variable strongly predicts a particular outbreak and dark colors (purple) indicating that an outbreak is more weakly predicted [30]. (Note: Color essential).

Shapley values showed varying contributions of different co-variates in predicting risks of different outbreak types (Figure 1). Contributions of frugivore richness for instance was high in outbreak predictions around Congo and Gabon (Figure 1a). LOOCV also corroborated this finding (Supplementary Materials), with very high predicted probabilities of spillover risk for outbreaks in Congo and Gabon. Frugivore richness predicted high probability of risk for all epizootic outbreaks and some human outbreaks centered in Gabon, Congo, and the Democratic Republic of Congo (Supplementary Materials shows LOOCV results). Contributions were relatively lower for outbreaks in Sudan, Uganda, and Ivory Coast (Figure 1a). Similarly, hunting had varying contributions to past outbreaks, with moderate to high contributions for outbreaks in eastern Democratic Republic of Congo, Uganda, Sudan, and Guinea (Figure 1b). With LOOCV, hunting activity predicted Sudan and Ivory Coast (now called Taï Forest) outbreaks, with moderate to high risk (probability >0.5) as well. However, it did predict outbreaks in eastern Gabon and Congo, including several *Zaire orthoebolavirus* outbreaks, with equal reliability (Supplementary Materials). Most outbreaks were predicted to some degree by *Ficus* suitability (Figure 1c). This pattern was more pronounced with LOOCV, where *Ficus* habitat suitability predicted all outbreaks with moderate to high probability (probability >0.5) (Supplementary Materials).

**Figure 2.**
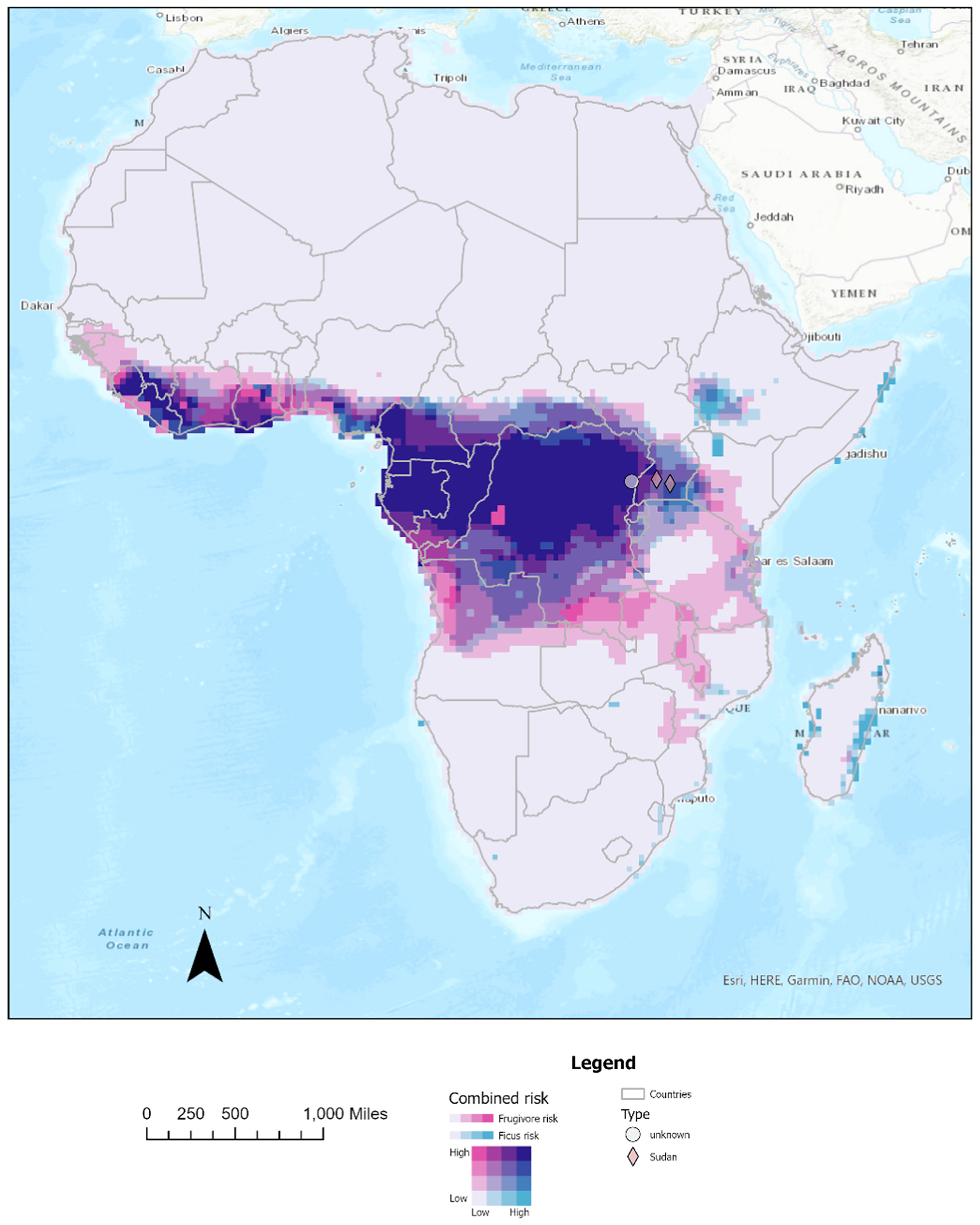
Bivariate risk map reflecting risk of *Orthoebolavirus* outbreaks from frugivore richness and *Ficus* habitat suitability. These two variables were chosen primarily because Sundaram et al. [16] showed that they were the two factors that most directly influenced spillover risk and had extremely high area under curve scores in our analyses (Table 1). Colors show where one or both variables predict relatively high spillover risk (e.g. with dark purple indicating cells with high risk according to both variables). Symbols indicate recent spillover events that were not included in analyses. (Note: Color essential).

Combined bivariate risk map of frugivore richness and *Ficus* habitat suitability suggests joint high risk of orthoebolaviruses in sub-Saharan Africa, as well as several areas of high risk from just one driver. Forested portions of central Africa and Western Africa show high joint risk of outbreaks resulting from high frugivore richness and high *Ficus* habitat suitability (Figure 2). In addition, some portions of eastern Madagascar show a high risk of *Orthoebolavirus* outbreaks but this largely due to *Ficus* availability (Figure 2). Areas of greater risk from *Ficus* overlap some past outbreaks of the Sudan virus type and are predicted to occur in portions of Uganda and Kenya (Figure 2). Areas of risk overlap recent outbreak events from 2022 to present (Figure 2). We provide individual driver predictions maps in supplementary material documents.

## Discussion

Our study sheds important insights into the conditions surrounding past *Orthoebolavirus* outbreaks. Past animal outbreaks or epizootics appear to be largely driven by frugivore richness and *Ficus* habitat suitability (Figure 1a). In addition, some human outbreaks of *Orthoebolavirus sudanense* are driven by human hunting activity and *Ficus* availability (Figures 1b and c). Using Shapley values and LOOCV, we demonstrate how the influence of different variables on past events can be characterized, even with limited outbreak data. Finally, we developed a bivariate map for spillover risk of many different types of *Orthoebolavirus* outbreaks (Figure 2).

Our analyses support a transmission mechanism in which frugivorous mammals facilitate viral spread and contribute to spillover events. We determined which variables were most important in transmission by ranking covariates based on their individual AUC values in distinguishing spillover from non-spillover locations in individual models. This is a non-traditional but novel use of AUCs to evaluate predictor importance across single-variable models. From this analysis, frugivore richness and richness of Cercopithecidae (the most speciose group of frugivorous primates in Africa) were the top overall predictors of spillover based on their individual AUC values, demonstrating strong discriminatory power between spillover and non-spillover locations (Table 1). Primates from the family Cercopithecidae have high mortality rates from exposure [15,31]. Overall, these results support the hypothesis that secondary amplifying hosts are important in transmission of orthoebolaviruses to human populations, whereas bats are the probable reservoir spreading the pathogen at sites of fruit consumption [16]. *Ficus* habitat suitability was also a top predictor of spillover, likely reflecting ecological conditions that promote frugivore aggregation. Fig stands have been hypothesized to be locations where animals gathered and developed infections [32]. Our machine learning models provide additional quantitative support for this idea. We found that *Ficus* habitat suitability was a strong predictor of *Orthoebolavirus* spillover (Table 1), which is also confirmed by path analyses [16]. Alternative theories, such as geographic variation in infected animal richness and abiotic conditions, were also tested as putative predictors of *Orthoebolavirus* outbreaks. However, these variables were not among the top covariates (Table 1), again suggesting that frugivorous mammals gathering around fruit resources, such as *Ficus*, are more important in driving outbreaks. Despite the strong support for frugivores in virus spread, we found that past outbreaks were mostly explained by *Ficus* availability and, to different degrees, by frugivore richness.

Our results also showed that different classes of *Orthoebolavirus* outbreaks are predicted to varying degrees by different underlying factors. Almost all of the outbreaks we considered seemed to be driven to some extent by spread of virus around fruit resources and possibly through interactions with frugivorous mammals (Figure 1). Shapley contributions and LOOCV indicated that all known past outbreaks could be predicted based on *Ficus* habitat suitability (Figure 1c, Supplementary Materials Figure S1). However, not all spillover events were driven by frugivore richness (Figure 1a). Particularly, epizootic outbreaks are predicted well by areas of frugivore richness (Figure 1a), which suggests that interactions among frugivorous mammals species are important in driving these events, which have led to severe declines in some ape populations historically [31,33]. Detailed data on when and how different mammals share fig fruit resources is likely critical to developing more accurate risk maps for non-human outbreaks (see Figure S2 for risk map based only on frugivore richness). For human outbreaks, proximity to fruiting figs may still play a role in exposure to orthoebolaviruses (Figure 1c).

Previous works have attempted to quantify triggers of orthoebolaviruses and spatiotemporal drivers of outbreaks [5,12]; however, limited understanding of spillover mechanisms has been a major obstacle. The absence of a clear reservoir species and other underlying factors involved in spread has historically created a lot of unknowns in defining spatial distributions from where the disease may be contracted. A recent study compared statistical support for a large number of path diagrams representing a variety of hypotheses about spillover mechanisms [16]. The best supported path diagrams suggested that the geographic distributions of frugivores around fruiting trees such as *Ficus* are important sources of spread of *Orthoebolavirus* infection. Yet, these conditions of frugivorous mammals gathering around fruiting resources had not been incorporated into spillover models and risk maps before this study.

Despite the overall importance of fruit resources and frugivorous secondary amplifying hosts from case history records [1], we know that not all outbreaks in human populations were initiated by people coming into contact with frugivorous mammals. For example, several *Orthoebolavirus sudanense* outbreaks may have started from insectivorous bats in the genera *Mops* [1,34]. Although most of the literature agree that Pteropodidae fruit bats are likely reservoirs of *Orthoebolavirus* (reviewed in [15]), the conundrum still remains of how insectivorous bats have tested positive for infections [35] and how these bats may have been at the epicenter of past outbreaks. There may be some spatiotemporal overlap between fruit bats and insectivorous bats in areas of high *Ficus* habitat suitability that scientists are yet to uncover. Certainly, *Ficus* suitability predicts all outbreaks in Sudan with high risk (Figure 1c), which suggests that overlap with fruit resources might be important for transfer of viruses. In addition, wild animal hunting activity also predicts Sudanese virus outbreaks (Figure 1b), which suggests that anthropogenic influences could play a role in these outbreaks. Case histories of some *Orthoebolavirus sudanense* outbreaks can be traced back to wild animal consumption, for instance, a 2004 outbreak in Yambio where the index case ate hunted *Papio anubis* [1]. However, at least one outbreak started in a factory infested with *Mops trevori* [36] and did not result from hunting [1], although our leave-one-out method suggests a high risk of spillover from this factor (Figure 1b). Hunting activity also does not predict some outbreaks in Congo, Mbomo, and Etoumbi regions specifically, and the descriptive case histories of these outbreaks suggest hunting inconclusively as the driver [1]. We suggest that hunting activity is certainly an important mechanism for spillover but that hunting activity alone may be too simple of a lens through which to view risk. For example, gathering of fruit resources in the same areas as other mammal species could also be an important risk factor, regardless of whether hunting occurs. Although a large amount of human exposure and many previous outbreaks can be predicted by data layers summarizing hunting activity, incorporating data on other socioeconomic factors, such as forest encroachment in general or poverty, which presumably drives some behaviors that may put individuals at risk, likely remains critical [21]. Latent human infections and subsequent resurgences through sexual transmission have also been identified as a mechanism for some more recent *Orthoebolavirus* outbreaks. Although this study was focused only on zoonotic spillover events, we have explored these latent mechanisms and their broader implications elsewhere [8].

The *Orthoebolavirus* case is a multivariate problem with few observations, which makes the development of a single risk map challenging. Given that epizootic outbreaks are driven by frugivore richness more compared with some human outbreaks and that hunting activity accounts for only some outbreaks, creating one risk map becomes challenging. In addition, the availability of just a few outbreaks makes it difficult to create separate but unique models for each outbreak type. Under these circumstances, data augmentation can be used as a tool to shed light on risks of different types of outbreaks. By running models with all pathogenic orthoebolaviruses, shared covariates of importance, such as *Ficus* habitat suitability, can be discovered. Further the relative importance of each driver in individual outbreaks can be tested *post hoc* using LOOCV methods or Shapley values. Because different variables influence types of *Orthoebolavirus* outbreak differently, a multivariate risk map is needed. We created a bivariate risk map from univariate risk due to frugivore richness and risk due to *Ficus* habitat suitability (Figure 2). Joint risk from both drivers likely show-cases areas where zoonotic and some human outbreaks occur. Areas with more risk due to *Ficus* (higher blue in Figure 2) can also increase risk of the Sudan-type viral outbreaks. We provide univariate risk maps from frugivore richness, *Ficus* habitat suitability, and wild animal hunting activity in the Supplementary Materials. Despite these uses of data augmentation, it is also important to note some limitations. Rigorous cross-validation of models and risk maps presented here is essential to ensure the reliability of data augmentation approaches before broader application. In addition, the proposed roles of frugivory and fruit resources, such as figs, in transmission warrants further empirical investigation to validate our findings. Until more data and confirmation are available, it could be argued that models trained on relatively low numbers of *Orthoebolavirus* outbreaks ([5,10,12,16,18,19], and this study) are somewhat exploratory.

Finally, the data limitations present in *Orthoebolavirus* occur in many disease systems. For example, the recent Rwandan outbreak of *Orthomarburgvirus* [37] brought the total number of known outbreaks to approximately 14 [38]. Data limitations are especially common in emerging infectious diseases [39]. In such study systems, we suggest that machine learning approaches leveraging Shapley values and LOOCV provide a useful approach for testing whether events in different regions or events that vary among other dimensions are yet adequately explained by a set of predictor variables.

To summarize, our study highlights the importance of the fruit-frugivore connection in *Orthoebolavirus* spread in sub-Saharan Africa. We show how fruit resources, namely, figs, predict locations of all past outbreaks with high accuracy. Zoonotic outbreaks in particular are associated with frugivore richness, and hunting activity predicts only some outbreaks, such as Sudan-type viruses. We advocate for a multivariate risk map and provide a predictive risk map of orthoebolaviruses for Africa.

## Supplementary Material

1

Supplementary material associated with this article can be found, in the online version, at doi:10.1016/j.ijid.2025.108180.

## Figures and Tables

**Table 1:** Performance metrics of machine learning models for every individual predictor of *Orthoebolavirus* spillover.

Predictor	Min AUC	Mean AUC	Max AUC	Sens Mean	Spec Mean
All mammals	0.779	0.909	0.996	0.805	0.845
*Non-bat mammal clades*					
Bovidae	0.534	0.737	0.921	0.641	0.751
Cervidae	0.500	0.500	0.504	0.000	1.000
Cercopithecidae	0.841	0.948	0.993	0.883	0.911
Hominidae	0.444	0.880	0.989	0.890	0.850
*Dietary specialization*					
Frugivores (60%≥)	0.791	0.954	0.998	0.875	0.932
Plant (60%≥)	0.097	0.708	0.976	0.618	0.685
Seed (60%≥)	0.128	0.661	0.872	0.499	0.887
Scavengers (60%≥)	0.442	0.784	0.847	0.643	0.925
*Bat clade*					
Pteropodidae	0.757	0.937	0.995	0.834	0.959
Nycteridae	0.852	0.930	0.979	0.856	0.949
Vespertilionidae	0.643	0.864	0.991	0.760	0.832
Hipposideridae	0.576	0.875	0.991	0.813	0.750
Rhinolophidae	0.547	0.772	0.950	0.746	0.642
Molossidae	0.560	0.847	0.994	0.752	0.795
*Ficus variables*					
Global *Ficus* habitat suitability	0.871	0.940	0.990	0.853	0.995
Local Ficus habitat suitability	0.580	0.797	0.951	0.616	0.913
Ficus richness	0.794	0.931	0.996	0.828	0.932
Ficus fruit volume	0.596	0.797	0.961	0.721	0.907
*Reservoir characteristics*					
Prop never infected	0.252	0.487	0.743	0.454	0.589
Prop fights infection	0.225	0.494	0.740	0.471	0.657
Prop dies and infected	0.157	0.578	0.876	0.524	0.601
Prop never encountered	0.211	0.493	0.780	0.453	0.563
Never infected	0.707	0.886	0.994	0.782	0.867
Fights infection	0.600	0.781	0.984	0.668	0.852
Dies and infected	0.752	0.891	0.985	0.804	0.882
Never encounters virus	0.786	0.916	0.998	0.822	0.871
*Hunting activity*					
Wild animal hunting (mean)	0.595	0.874	0.988	0.859	0.714
Wild animal hunting (median)	0.360	0.841	0.988	0.878	0.618
*Environmental factors*					
Mean annual temperature	0.216	0.493	0.757	0.470	0.609
Temperature seasonality	0.816	0.912	0.987	0.812	0.957
Annual precipitation	0.827	0.924	0.983	0.833	0.933
Precipitation seasonality	0.641	0.802	0.964	0.690	0.828

Ensemble logistic regression models were fitted predicting spillover (1) vs no spillover (0) for each predictor with repeated cross-validation and downsampling. For each predictor, we summarize the minimum AUC, mean AUC, and maximum AUC across different resampled models from repeated cross-validation process. We also summarize mean sensitivity (Sens Mean) and mean specificity (Spec mean). Variables were chosen based on factors hypothesized to drive or predict spillover risk in previous studies.
