# Supplementary material for: Spatial risks of Orthoebolavirus spillover vary based on outbreak type
Source: Int J Infect Dis. Author manuscript; Available in PMC 2025 Dec 15. (PMC12705173; doi:10.1016/j.ijid.2025.108180)
Supplement: 1 [file NIHMS2126051-supplement-1.docx]

**Supplementary Materials**


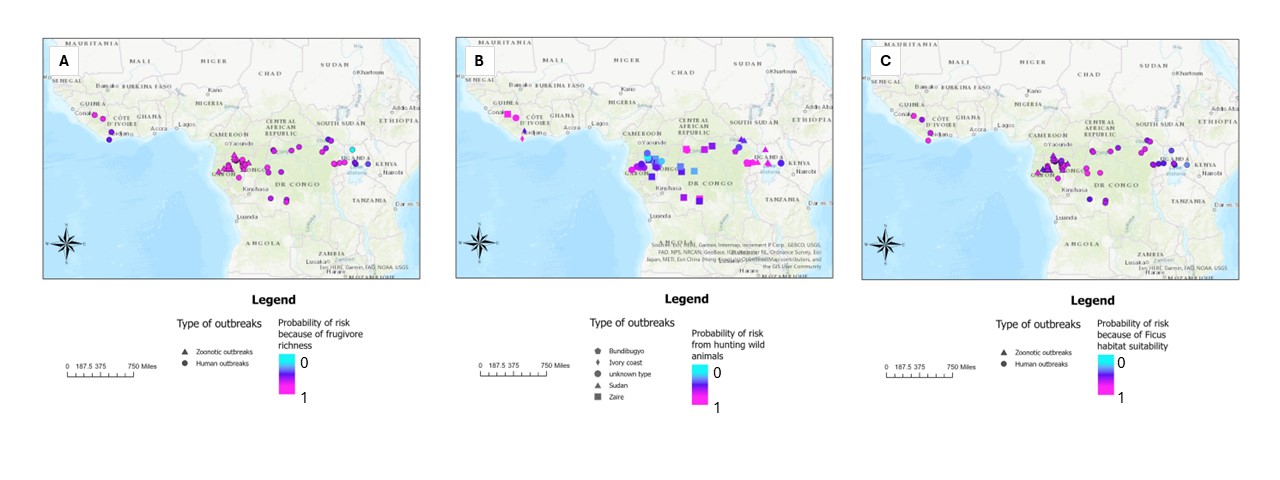


Figure S1. Predictive accuracy of important covariates in explaining all past *Orthoebolavirus* outbreaks using Leave-one-out cross validation (LOOCV) method. Risk of zoonotic and human outbreaks determined using LOOCV predicted by frugivore richness (A). Risk of *Orthoebolavirus* outbreak types determined using LOOCV predicted by wild animal hunting activity (B). Risk of zoonotic and human outbreaks determined using LOOCV predicted by *Ficus* habitat suitability (C).


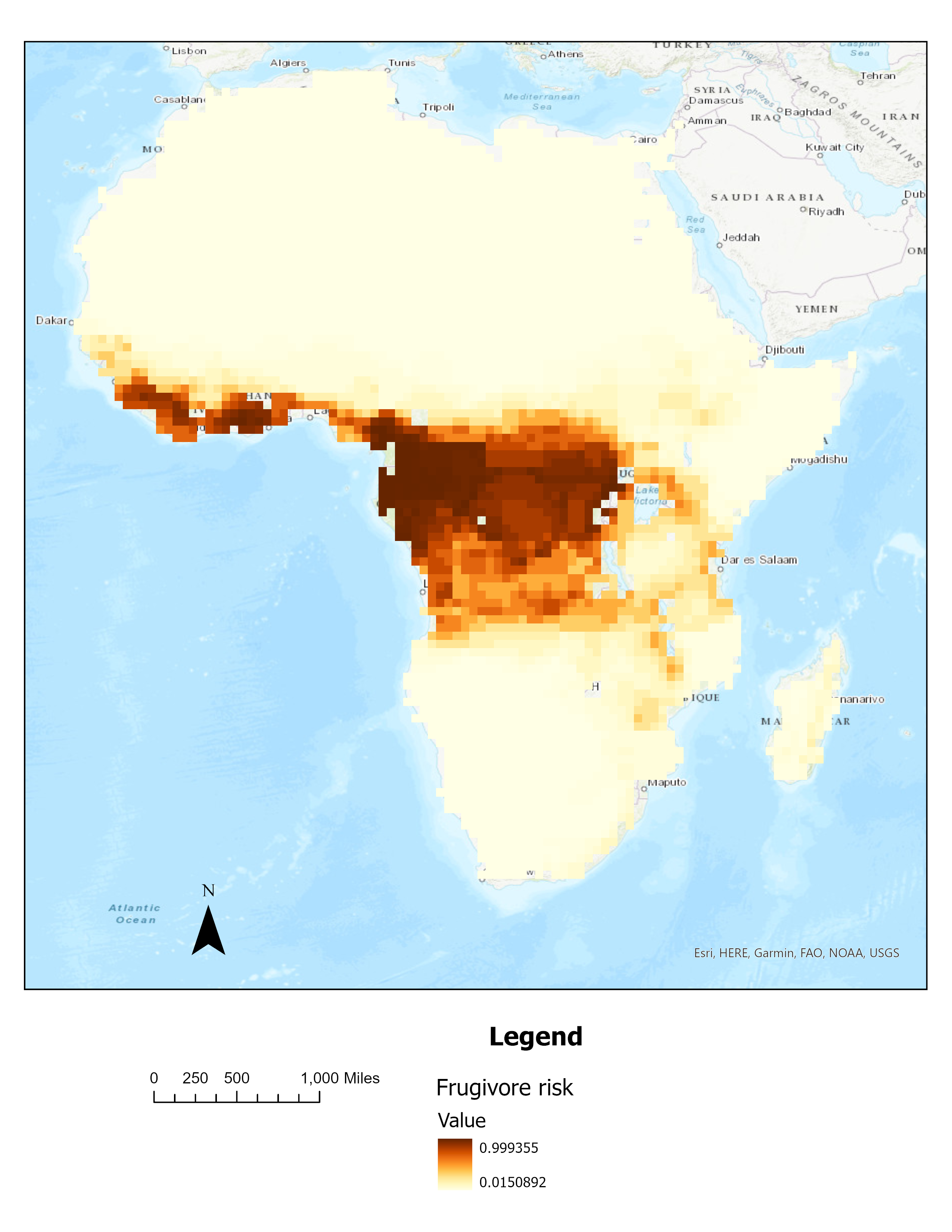


Figure S2. Univariate risk map reflecting risk of *Orthoebolavirus* outbreaks from frugivore richness


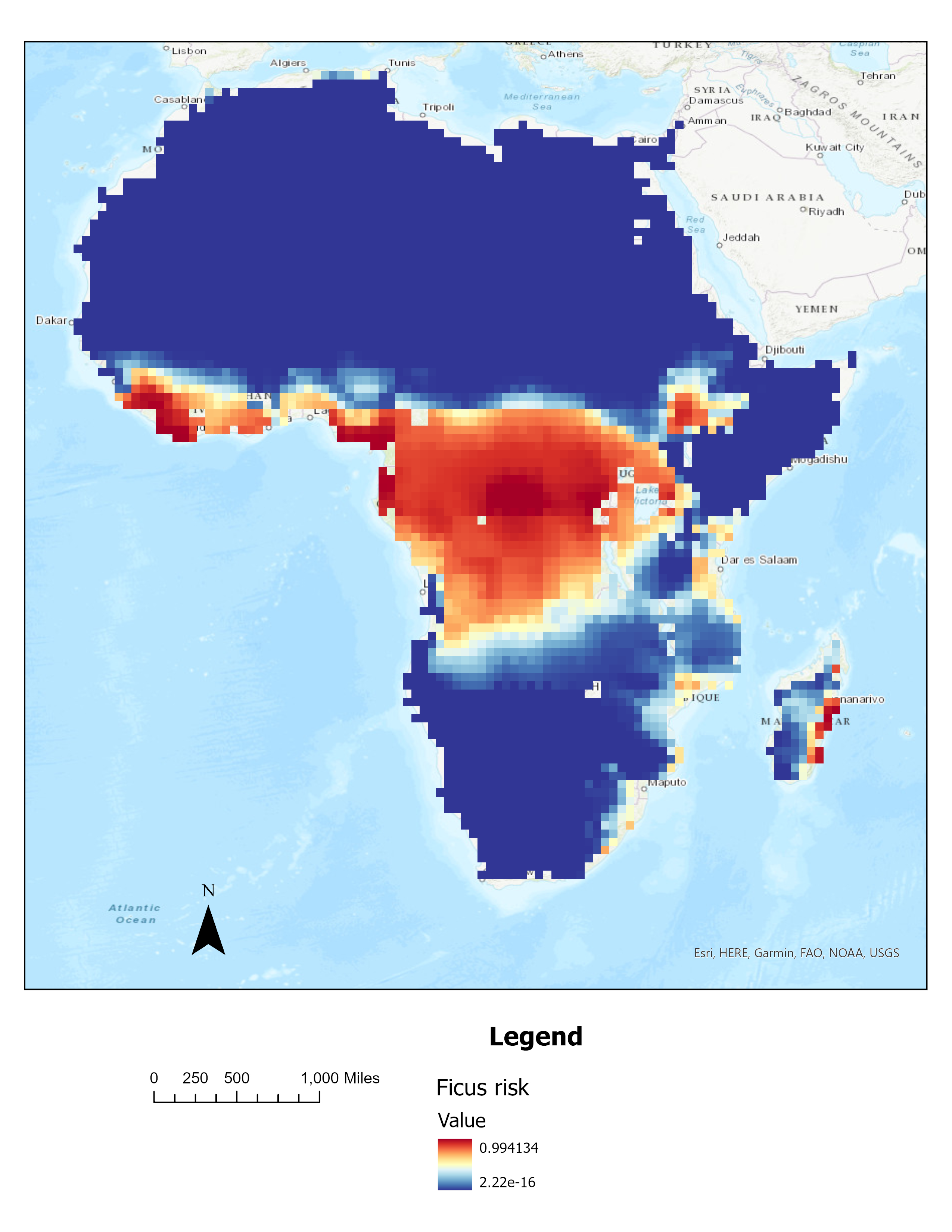


Figure S3. Univariate risk map reflecting risk of *Orthoebolavirus* outbreaks from *Ficus* habitat suitability.


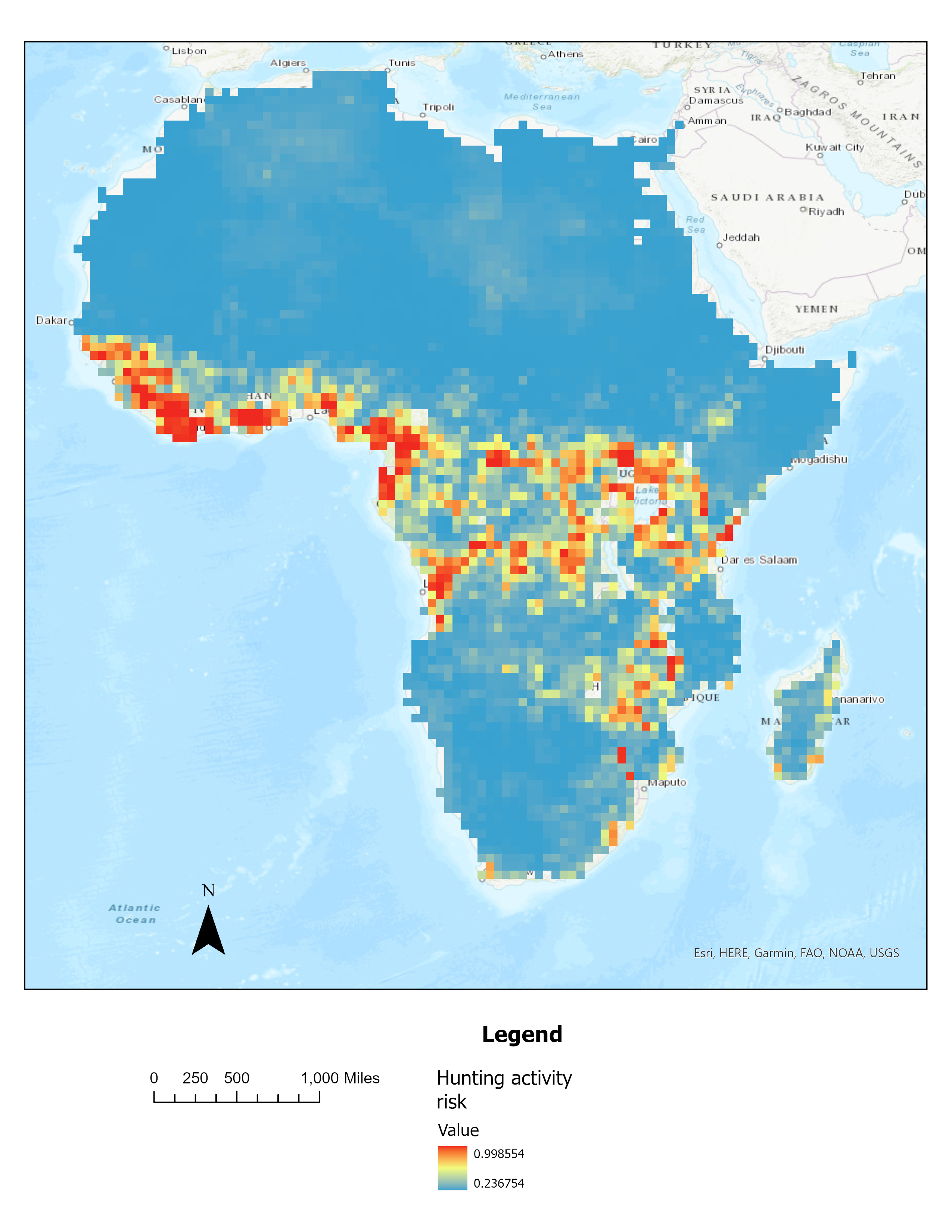


Figure S4. Univariate risk map reflecting risk of *Orthoebolavirus* outbreaks from wild animal hunting activities.

Table S1. *Orthoebolavirus* outbreaks from 2022 to present. Each row provides details of outbreak events, including its place of origin, georeferenced longitude and latitude, type of virus responsible for the outbreak and reference describing outbreak.

| Place of origin | Longitude | Latitude | Type | Reference |
| --- | --- | --- | --- | --- |
| Butsili-Beni | 29.4453979 | 0.50180273 | Ebola (no reference to species) | https://africacdc.org/news-item/the-democratic-republic-of-congo-declares-over-the-thirteenth-ebola-virus-disease-outbreak/ |
| Kampala | 32.581111 | 0.313611 | Sudan | https://www.who.int/emergencies/disease-outbreak-news/item/2025-DON566 |
| Ngabano Village | 31.4807 | 0.6844 | Sudan | https://pmc.ncbi.nlm.nih.gov/articles/PMC11413514/#:~:text=On%20Sept%2020%2C%202022%2C%20the%20Ministry%20of,disease%20outbreak%20in%20Mubende%20District%2C%20Central%20Uganda.&text=On%20Sept%2019%2C%202022%2C%20a%20blood%20sample,virus%20at%20the%20Uganda%20Virus%20Research%20Institute. |
